# COVID-19 vaccine perceptions in the initial phases of US vaccine roll-out: an observational study on reddit

**DOI:** 10.1186/s12889-022-12824-7

**Published:** 2022-03-07

**Authors:** Navin Kumar, Isabel Corpus, Meher Hans, Nikhil Harle, Nan Yang, Curtis McDonald, Shinpei Nakamura Sakai, Kamila Janmohamed, Keyu Chen, Frederick L. Altice, Weiming Tang, Jason L. Schwartz, S. Mo Jones-Jang, Koustuv Saha, Shahan Ali Memon, Chris T. Bauch, Munmun De Choudhury, Orestis Papakyriakopoulos, Joseph D. Tucker, Abhay Goyal, Aman Tyagi, Kaveh Khoshnood, Saad Omer

**Affiliations:** 1 Section of Infectious Diseases, Yale School of Medicine, New Haven, CT USA; 2 Yale College, New Haven, CT USA; 3 Department of Biostatistics, Yale School of Public Health, New Haven, CT USA; 4 Department of Statistics, Yale University, New Haven, CT USA; 5 Department of Epidemiology of Microbial Diseases, Yale School of Public Health, New Haven, CT USA; 6 University of North Carolina Project-China, Guangzhou, China; 7 Social Entrepreneurship to Spur Health (SESH) Global, Guangzhou, China; 8 University of North Carolina at Chapel Hill, Chapel Hill, NC USA; 9 Department of Health Policy and Management, Yale School of Public Health, New Haven, CT USA; 10 Department of Communications, Boston College, Boston, MA USA; 11 Microsoft Research Lab, Montreal, Québec Canada; 12 New York University, Abu Dhabi, UAE; 13 Department of Applied Mathematics, University of Waterloo, Waterloo, Ontario Canada; 14 School of Interactive Computing, Georgia Tech, Atlanta, GA USA; 15 Center for Information Technology Policy, Princeton University, Princeton, NJ USA; 16 School of Medicine, University of North Carolina at Chapel Hill, Chapel Hill, NC USA; 17 Faculty of Infectious and Tropical Diseases, London School of Hygiene and Tropical Medicine, London, USA; 18 Department of Computer Science, Stony Brook University, New York, NY USA; 19 Engineering and Public Policy, Carnegie Mellon University, Pittsburgh, PA USA; 20 Yale Institute for Global Health, New Haven, CT USA

**Keywords:** COVID-19, Vaccine, Reddit, Computational, Misinformation

## Abstract

**Background:**

Open online forums like Reddit provide an opportunity to quantitatively examine COVID-19 vaccine perceptions early in the vaccine timeline. We examine COVID-19 misinformation on Reddit following vaccine scientific announcements, in the initial phases of the vaccine timeline.

**Methods:**

We collected all posts on Reddit (reddit.com) from January 1 2020 - December 14 2020 (n=266,840) that contained both COVID-19 and vaccine-related keywords. We used topic modeling to understand changes in word prevalence within topics after the release of vaccine trial data. Social network analysis was also conducted to determine the relationship between Reddit communities (subreddits) that shared COVID-19 vaccine posts, and the movement of posts between subreddits.

**Results:**

There was an association between a Pfizer press release reporting 90% efficacy and increased discussion on vaccine misinformation. We observed an association between Johnson and Johnson temporarily halting its vaccine trials and reduced misinformation. We found that information skeptical of vaccination was first posted in a subreddit (r/Coronavirus) which favored accurate information and then reposted in subreddits associated with antivaccine beliefs and conspiracy theories (e.g. conspiracy, NoNewNormal).

**Conclusions:**

Our findings can inform the development of interventions where individuals determine the accuracy of vaccine information, and communications campaigns to improve COVID-19 vaccine perceptions, early in the vaccine timeline. Such efforts can increase individual- and population-level awareness of accurate and scientifically sound information regarding vaccines and thereby improve attitudes about vaccines, especially in the early phases of vaccine roll-out. Further research is needed to understand how social media can contribute to COVID-19 vaccination services.

## Introduction

The first vaccine against COVID-19 from Pfizer-BioNTech received emergency use authorization (EUA) on December 11, 2020 [1]. On August 23, 2021, The US FDA issued a Letter of Approval to Pfizer for full use of the vaccine, under the name COMIRNATY for use in persons aged 16 years and older and delayed pediatric approval for those younger than 12 years [2]. From early 2021 on, COVID-19 vaccines have helped alleviate the pandemic’s burden on society by mitigating contagion, protecting the population against severe disease, and allowing for less restrictive measures, especially in countries with high vaccine uptake and availability [3]. Since mid-2021, vaccine availability does not pose a problem in high-income countries. Instead, these countries face the challenge that vaccine-hesitant and vaccine-denial individuals pose to the timely completion of vaccination programs [4]. There are a diverse range of individuals who are skeptics of vaccination including those who are antivaccine or antivaxxers (individuals who are opposed to vaccination or laws that require vaccination) and those who are vaccine hesitant (those who delay in acceptance or refusal of vaccination) [5]. The diverse groups who are skeptical of vaccines may react to information in different ways [6]. Often, the lack of individuals willing to receive the vaccine at a given moment has caused the expiration and discard of available vaccine doses [7]. Therefore, it is crucial for these countries, especially the US, to understand the drivers of vaccine hesitancy [8] and implement timely initiatives for re-selling or donating surplus doses to countries where they are needed. More recently, a new wave of COVID-19 cases caused by the highly transmissible delta and omicron variants is exacerbating the worldwide public health crisis, and has led to consideration of the potential need for, and optimal timing of, booster doses for vaccinated populations [9].

Thus, for these vaccines to be successful, they not only need to be deemed safe and effective by scientists, but also widely accepted by the public [10]. Effective health communication is key to vaccine acceptance, but is a complex task given widespread vaccine hesitancy, rapidly changing vaccine information [11], and vaccine misinformation [12]. Vaccine hesitancy is the reluctance of people to receive safe and recommended available vaccines, already a growing concern before the COVID-19 pandemic [11]. Vaccine hesitancy results from a complex decision-making process, influenced by a wide range of contextual, individual and group, and vaccine-specific factors, including communication and media, historical influences, religion/culture/gender/socioeconomic status, politics, geographic barriers, experience with vaccination, risk perception, and design of the vaccination program [13]. Misinformation is defined as information that has the features of being false, determined based on expert evidence, but shared with no intention of harm [14]. Such information may worsen existing fear around a vaccine and limit public uptake of a COVID-19 vaccine and its boosters [12]. With low willingness to vaccinate globally [15], and substantial COVID-19 misinformation [16], achieving sufficient vaccination coverage to reach population-level benefits will be challenging.

Reduced vaccine uptake may impinge on population-level impact [17], and COVID-19 control at the population level [4]. For example, reduced vaccine uptake may increase the mortality cost of COVID-19 [18] and create clusters of non-vaccinators that disproportionately increase pandemic spread [19]. In addition, willingness to accept a COVID-19 vaccine seems to be fluctuating in the US [20]. Thus, vaccine acceptance is not constant or uniform, and likely affected by several factors, such as being responsive to information and perceptions regarding the vaccine, and the state of the pandemic and economy.

Several studies have detailed the relationship between exposure to COVID-19 misinformation and vaccine acceptance [21], as well as COVID-19 vaccine perceptions assessed via Twitter [22, 23] and online surveys [24, 25]. However, there are very few articles that explore at the influence of the peer-groups on COVID-19 vaccination decisions. For example, recent work verified whether there is a strong correlation between the pro-vaccination, against COVID-19 attitude of the respondents and their belief that most of those around them want to be vaccinated against COVID-19 [26]. There has been limited work that explores how online peer-groups relate to COVID-19 vaccinations. Similarly, relatively few studies have focused on Reddit (reddit.com), a social news aggregation and discussion website. Registered Reddit members submit posts (text, images, videos) to the site, which are then voted up or down by other members. Posts are organized by subject into user-created boards called communities or subreddits, which cover a large range of topics. Reddit may be a useful setting for examining vaccine perceptions because similar topics have been discussed before [27], including topics related to COVID-19 vaccine development [28]. Moreover, as seen with the recent GameStop trading event, Reddit is increasingly important in online conversations [29]. We note that Reddit and similar online sources are not necessarily representative of what the overall US general public feels [30]. However, Reddit provides insights on highly shared news, and can rapidly transmit both misinformation and accurate information [31–33].

Recent work observed how the HPV vaccine is characterized on Reddit over time and by user gender. Findings demonstrated that women and men both discussed HPV, highlighting that Reddit users do not perceive HPV as an issue that only pertains to women [34]. A similar study indicated that Reddit users perceived the HPV vaccine domain from a virus-framed perspective that could impact their lifestyle choices and that their awareness of the HPV vaccine for cancer prevention is also lacking [35]. Regarding COVID-19, researchers used sentiment analysis and topic modeling on data collected from Reddit communities focusing on the COVID-19 vaccine from Dec 1, 2020, to May 15, 2021, finding that sentiments expressed in these communities are overall more positive than negative and have not meaningfully changed since December 2020 [36]. Another study used topic modelling to generate latent topics from user generated Reddit corpora on reasons for vaccine hesitancy, finding factors such as fear of risks and side effects, and lack of trust in policymakers [37]. A study using COVID-19 Reddit data and topic modelling found that during the pandemic, the proportion of Reddit comments predominated by conspiracy theories outweighed that of any other topics [38]. However, limited research has explored how online vaccine perceptions are associated with major events in the early in the vaccine development and implementation timeline (e.g. major pharmaceutical firms halting vaccine trials or publishing results on vaccine effectiveness) and how online vaccine discussions move across arenas that have different baseline vaccine perceptions.

We thus propose a study to detail the behavior of top Reddit users, posts’ relationship with events in the initial phases of vaccine timeline, and the relationship between subreddits that shared COVID-19 vaccine posts. We provide an overview of Reddit conversations around the COVID-19 vaccine from January 1 2020 - December 14 2020, focusing on everyone who shared COVID-19 vaccine posts in English, to give understanding of vaccine narratives when vaccines were first trialed and introduced. It is important to understand the behavior of top users, how vaccine perceptions are related to events in the vaccine timeline and how vaccine discussion on Reddit migrates across subreddits that differ in their vaccine perceptions, to mitiate vaccine misinformation early in the vaccine development timeline. Most users of online platforms are passive or participate with a very low frequency. A small number of Reddit users are hyperactive and may over-proportionally influence vaccine perceptions online [39]. Thus, describing the behavior of hyperactive users is key to understanding shifts in vaccine perceptions, early in the vaccine timeline. Understanding how perceptions are related to intital vaccine-related events may allow stakeholders to better design communication and education campaigns [40, 41] in response to early vaccine distribution setbacks. Given the range of vaccine-related viewpoints online, greater insight on how discussions move across Reddit communities will allow stakeholders to better disseminate evidence-based information on Reddit. The purpose of this analysis was to detail the behavior of top Reddit users, posts’ relationship with events early in the vaccine timeline, and the relationship between subreddits that shared COVID-19 vaccine posts. Research questions are as follows: What is the behavior of top Reddit users in regards to COVID-19 vaccines? What are Reddit posts’ relationship with events early in the vaccine timeline? What is the relationship between subreddits that shared COVID-19 vaccine posts? Our findings hope to inform stakeholders on how to manage online narratives around vaccines early in the vaccine timeline, to mitigate misinformation as it arises. Developing vaccine misinformation mitigation techniques early in the vaccine timeline is critical to managing misinformation before it proliferates later in the vaccine timeline.

## Methods

### Data acquisition and processing

Using the Pushshift API and the Python Reddit API Wrapper [42, 43], we collected all posts on the entire Reddit (reddit.com), across all subreddits from January 1 2020 - December 14 2020 that contained both COVID-19 and vaccine keywords (see Supplement, only posts that had COVID-19 AND vaccine-related keywords were collected) derived from systematic reviews on the topic. The Pushshift API was designed and created by the /r/datasets mod team to help provide enhanced functionality and search capabilities for searching Reddit comments and submissions. The API was used directly via api.pushshift.io. We used the q parameter to search for a specific word or phrase. Here is an example where we search for the most recent comments mentioning the word *vaccine* (api.pushshift.io/reddit/search/comment/?q=vaccine). This searched the most recent comments with the term *vaccine* in the body of the comment. This search is not case-sensitive, so it will find any occurence of the term *vaccine* regardless of capitalization. The API defaults to sorting by recently made comments first. Data was returned in JSON format. Reddit is a publicly available website. We also collected metadata for each post e.g. the username, ID, subreddit. We then preprocessed our data as follows: 1) removed duplicate entries; 2) filtered out entries <50 characters as these generally do not provide enough information for meaningful analysis [44, 45]; 3) filtered the content using a curated set of search terms (as shown in the Supplement) to retain only COVID-19 vaccine-related content; 4) removed text in non-English languages, URLs, emojis, and punctuation. Our data collection strategy centers our work on everyone globally who shared COVID-19 vaccine posts in English.

### Hyperactive users

To better understand the possibly outsize influence of some individuals, we provided a descriptive overview of the behavior of top 10 users, focusing on content and number of posts.

### Topic modeling

We used topic modeling to understand changes in word prevalence within topics around COVID-19 vaccines (see Supplement for additional detail). Topic modeling is a computer-aided content analysis technique through which texts are organized into themes known as “topics” [46, 47]. We used an approach to topic modeling known as Structural Topic modeling (STM) [48, 49]. STMs [48, 49] enable the generation of topics with regards to document metadata such as date and source and other covariates relevant to the research question, such as new COVID-19 cases, and thus was used instead of other topic modelling methods. We used the following metadata covariates for the STM model: date (1 was denoted for the first day and numbered sequentially after), new COVID-19 cases per day worldwide, new COVID-19 deaths per day worldwide (publicly available and both obtained from COVID-19 Data Repository by the Center for Systems Science and Engineering at Johns Hopkins University [50]), S and P 500 opening score (publicly available from the Wall Street Journal), post type (comment or post), score (upvotes - downvotes). We used worldwide cases and deaths instead of US cases/deaths as Reddit COVID-19 discussion centers on pandemic progression both globally and in the US, despite most users being from the US. These control variables may address underlying factors possibly influencing vaccine perceptions. By considering a broader picture of what may influence topic proportions around vaccine discussion, we can better test the claims relation to the association between specific events and topic proportions. March 11 2020 was denoted as the start date for our analysis, the date the World Health Organization declared COVID-19 a pandemic [51].

As STM is an unsupervised approach, the number of topics (k) to estimate is key to the analysis. We first estimated several models ranging from 5 to 30 topics. These models were then evaluated qualitatively by two authors (IC, AG) independently for 1) their ability to produce coherent topics and 2) appropriately capture topics regarding COVID-19 vaccination [52]. The two authors agreed on the same topic solution (k=20). Topic interpretation was influenced by authors’ first reading the top 100 most-cited COVID-19 peer-reviewed research articles and the top 10 most cited peer-reviewed research articles around topic modeling. Two authors assigned topics (IC, AG) [Cohen’s kappa (k) >0.8] and a third author (NK) resolved disagreements when they arose [Cohen’s kappa (k) >0.8].

**Fig. 1:**
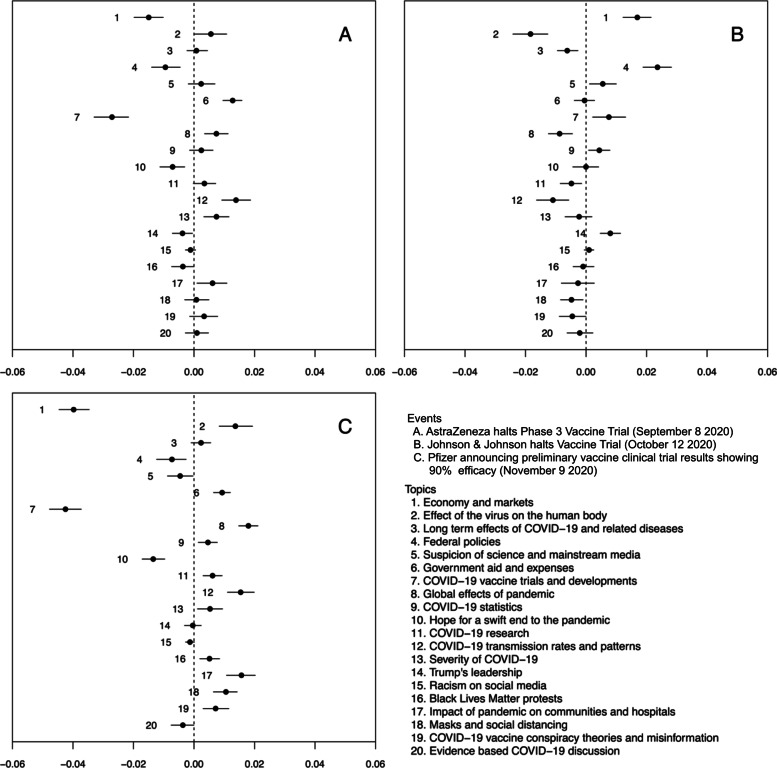
Regression where the outcome variable was the proportion of each document dedicated to each topic. Values were generated from a regression where the outcome variable was the proportion of each document dedicated to each topic, given the selected STM model, and with various vaccine events as main explanatory variables. Topics on the right of the zero line were more likely to be brought up after the indicated event. Confidence intervals (95%) included both regression uncertainty and measurement uncertainty from the STM model. **A**, **B**, and **C** refer to the above indicated events

We also detailed how events in the vaccine timeline (described in following section) were associated with topic prevalence. We generated linear regression models with expected topic proportions for each topic as dependent variables and vaccine events as main explanatory variables, with the following additional covariates: new COVID-19 cases per day worldwide, new COVID-19 deaths per day worldwide, S&P 500 opening score, post type. We first conducted a visual examination on the pattern of the time series by plotting them and generating auto-correlation and partial correlation plots. No seasonal patterns were identified. Auto-correlation was tested with the Durbin-Watson test. Nonstationarity was identified using the augmented Dickey-Fuller test and corrected through differencing. To validate regression analyses in Fig. 1, we undertook a close reading of the 100 most representative text fragments for exemplar topics. We found that these topics varied in line with the indicated events.

### Selecting events of interest

We used the following steps to assemble a preliminary list of COVID-19 vaccine-related events: 1) We selected three content experts who had published at least ten peer-reviewed articles in the last three years around vaccination. The content experts developed a list of ten key events separately through consulting online news sites and peer-reviewed vaccine research articles. 2) The three experts then discussed their lists to result in a final list of six events (see Supplement) that were broadly similar across all three original lists. We then conducted preliminary analyses with remaining events to determine the ones that were associated with the greatest shift in topic proportions for each topic. The three events below were selected as our final list, as these were associated with a shift in topic proportions for most topics. Events as follows: 1) AstraZeneca halts Phase 3 vaccine trial (September 8 2020); 2) Johnson & Johnson temporarily halts vaccine trial (October 12 2020); 3) Pfizer announces preliminary vaccine clinical trial results showing 90% efficacy (November 9 2020).

### Social network analysis

Next, we conducted social network analysis to provide insights on how vaccine discussion on Reddit migrates across subreddits that differ in their vaccine perceptions, and the relationship between these subreddits [53]. While standard social networks tend to assess relationships between people, we used a network to describe relationships between subreddits, studying the connections between people as mediated by the subreddits they were in and the posts shared between these subreddits. We used igraph [54] for social network analysis. All data was downloaded into a csv file. The csv file contained two columns with node information. For example, if the first column had node A and the second column had node B, this meant that the output would be A- >B, where A and B are connected nodes with A having a directed edge toward B. In our study, A and B both represented subreddits. Edge direction was based on whether a node had >50% of its posts made prior to its adjacent connecting node e.g. A- >B if >50% of A’s shared posts were made before B. When analyzing the trajectory of posts from one subreddit to another, we assumed that posts moved from A- >*B*−>C if a post was made first in A, then followed by B and C. This may allow us to see how posts moved from one subreddit to another. We passed this csv file to igraph, which provided a network diagram. We used node sizes to represent number of users in a subreddit, node edges to indicate shared COVID-19 vaccine posts between subreddits (an edge was indicated if there was >one shared post), and node labels to detail the subreddit name. We used the fast-greedy algorithm for cluster identification. The fast-greedy algorithm is an efficient approach to detect communities based on modularity. This strategy starts with a subnetwork composed only of links between highly connected nodes. Then, the algorithm iteratively samples random links that improve the modularity of the subnetwork and adds them. This iterative process is repeated as long as the modularity keeps improving. Finally, the communities are obtained based on the connected components in the subnetwork [55]. We focused on the main social network in our data (largest component subgraph) and excluded all edges with a weight of one (i.e. all connections between subreddits that had only one post in common) and all clusters that had <15 vertices and whose vertices had a betweenness centrality <20 (we used a range of network characteristics to yield an easy to understand social network and the above measures yielded the clearest output).

## Results

Post-processing, we had 266,840 documents (25,400,556 words).

### Overview of hyperactive users

We reviewed the posts for the top 10 users who posted the most in our dataset, ranging from 159 - 278 posts/person. Six of these users posted evidence-based information (e.g. Effectiveness of the COVID-19 vaccine: real-world evidence from healthcare workers, Vaccine linked to reduction in risk of COVID-19 admissions to hospitals), but four users (one of these users was suspended from Reddit at time of writing) seemed to be skeptical of vaccination (e.g. Hell Gates says Vaccines are Americans’ only hope to return to Normal Life!, Doctors Around the World Issue Dire Warning: DO NOT get the experimental covid vaccine, At What Point Do We Realize Bill Gates Is Dangerously Insane?). Individuals skeptical of vaccination were common among those who posted the most frequently in our data.

### Topic modeling

Table 1 indicated the topics in the dataset, their proportions, and the top 10 words for each topic (see Table 1). Broadly, our data centered on the severity of the pandemic (Topic 13), hope for a swift end to the pandemic (Topic 10), and suspicion of science and mainstream media (Topic 5). The severity of the pandemic topic focused on death, risk and sickness in relation to the pandemic. The hope for a swift end to the pandemic topic was about hope, safety and the length of the pandemic. Finally, the suspicion of science topic was around reduced trust and belief in the media and science. We also noted several other topics, such as evidence-based COVID-19 discussion (exploring factually sound and true evidence about COVID-19) (Topic 20), COVID-19 transmission rates and patterns (Topic 12), the effect of the virus on humans (Topic 2), COVID-19 vaccine conspiracy theories and misinformation (e.g. Bill Gates-related vaccine conspiracy theories) (Topic 19), and racism on social media (Topic 15).

**Table 1:** Structural topic model results from 266,840 documents, March 11 2020 - December 14 2020, including the topic proportion and the top 10 words associated with each topic

Expected Topic Proportions	Topics Title	Top 10 words
0.1036	Severity of COVID-19	die, risk, life, normal, live, sick, yes, stop, stay, serious
0.0941	Hope for a swift end to the pandemic	long, term, hope, safe, next, available, wait, shot, pretty, rush
0.0861	Suspicion of science and mainstream media	believe, anti, tell, real, science, liter, bad, media, cure, trust
0.0796	Evidence based COVID-19 discussion	read, fact, understand, inform, clear, true, person, reason, post, evidence
0.0702	COVID-19 transmission rates and patterns	spread, population, herd, high, risk, rate, number, reduce, hospitalization, mortality
0.0633	Effect of the virus on the human body	common, system, response, human, influenza, body, cold, mutate, strain, similar
0.0577	Impact of pandemic on communities and hospitals	home, school, family, care, person, stay, live, learn, hospital, help
0.0523	Global effects of pandemic	world, country, public, global, travel, economy, social, govern, open, state
0.0500	COVID-19 vaccine trials and developments	data, phase, trial, clinic, safety, studies, drug, efficacy, severe, receive
0.0478	COVID-19 statistics	death, million, rate, dead, season, die, total, number, second, near
0.0381	Economy and markets	market, companies, stock, product, industry, supply, price, sell, demand, billion
0.0376	Federal policies	trump, president, nation, state, elect, administration, federal, house, unit, response
0.0359	Government aid and expenses	govern, money, free, pay, business, cost, spend, economy, system, support
0.0325	Long term effects of COVID-19 and related diseases	medical, damage, cancer, doctor, heart, polio, measles, harm, medicine, child, blood
0.0310	Black Lives Matter protests	game, watch, video, police, fire, sport, street, post, show, red
0.0309	COVID-19 vaccine conspiracy theories and misinformation	bill, world, power, human, war, russia, mark, control, chip, conspiracy
0.0294	COVID-19 research	research, link, studies, found, scientific, respiratory, science, paper, associate, article
0.0291	Masks and social distancing	wear, mask, social, protect, spread, face, public, hand, person, distance
0.0160	Racism on social media	chance, please, remember, pass, remove, black, message, thank, stick, attend
0.0148	Trump’s leadership	march, trump, perfect, control, disappear, march, anybody, fine, false, great

Note: The topic proportions indicated the proportion of the corpus that belongs to each topic

We then explored how various events in the vaccine timeline were related to topic prevalence (see Fig. 1). We observed an association between AstraZeneca temporarily halting its vaccine trials, and increased discussion around government expenses (*β*_intercept_ = 5.019e-02, *p*<0.001), such as funds spent on businesses, and supporting the economy. Similarly, we found an association between Johnson and Johnson temporarily halting its vaccine trial, and increased discussion of federal policies (*β*_intercept_ = -1.08e-2) and then-US President Donald Trump’s leadership (*β*_intercept_ = 4.330e-02, *p*<0.001). We found an association among Johnson and Johnson temporarily halting its vaccine trial and greater discussion around suspicion of science and mainstream media (*β*_intercept_ = 7.536e-02, *p*<0.001). Similarly, there was an association between Pfizer announcing preliminary Phase 3 results showing 90% vaccine efficacy and reduced discussion about suspicion of science and mainstream media (*β*_intercept_ = 1.027e-01, *p*<0.001). We detailed an association between Johnson and Johnson temporarily halting its vaccine trial and reduced discussion around COVID-19 vaccine conspiracy theories and misinformation (*β*_intercept_ = 2.619e-02, *p*<0.05). We found an association between Pfizer announcing preliminary vaccine clinical trial results, an increase in discussion around COVID-19 vaccine conspiracy theories and misinformation (*β*_intercept_ = -1.11e-3), and a corresponding decrease in evidence-based COVID-19 discussion (*β*_intercept_ = 5.291e-02, *p*<0.001). We also found that new COVID-19 deaths and cases were positively associated with increased discussion around COVID-19 vaccine conspiracy theories and misinformation (*β*_intercept_ = 6.517e-07, *p*<0.01), and suspicion of science and mainstream media (*β*_intercept_ = 2.99e-7), highlighting the relationship between COVID-19 progression and similar rises in misinformation (See Supplement for full results).

### Subreddit networks

**Fig. 2:**
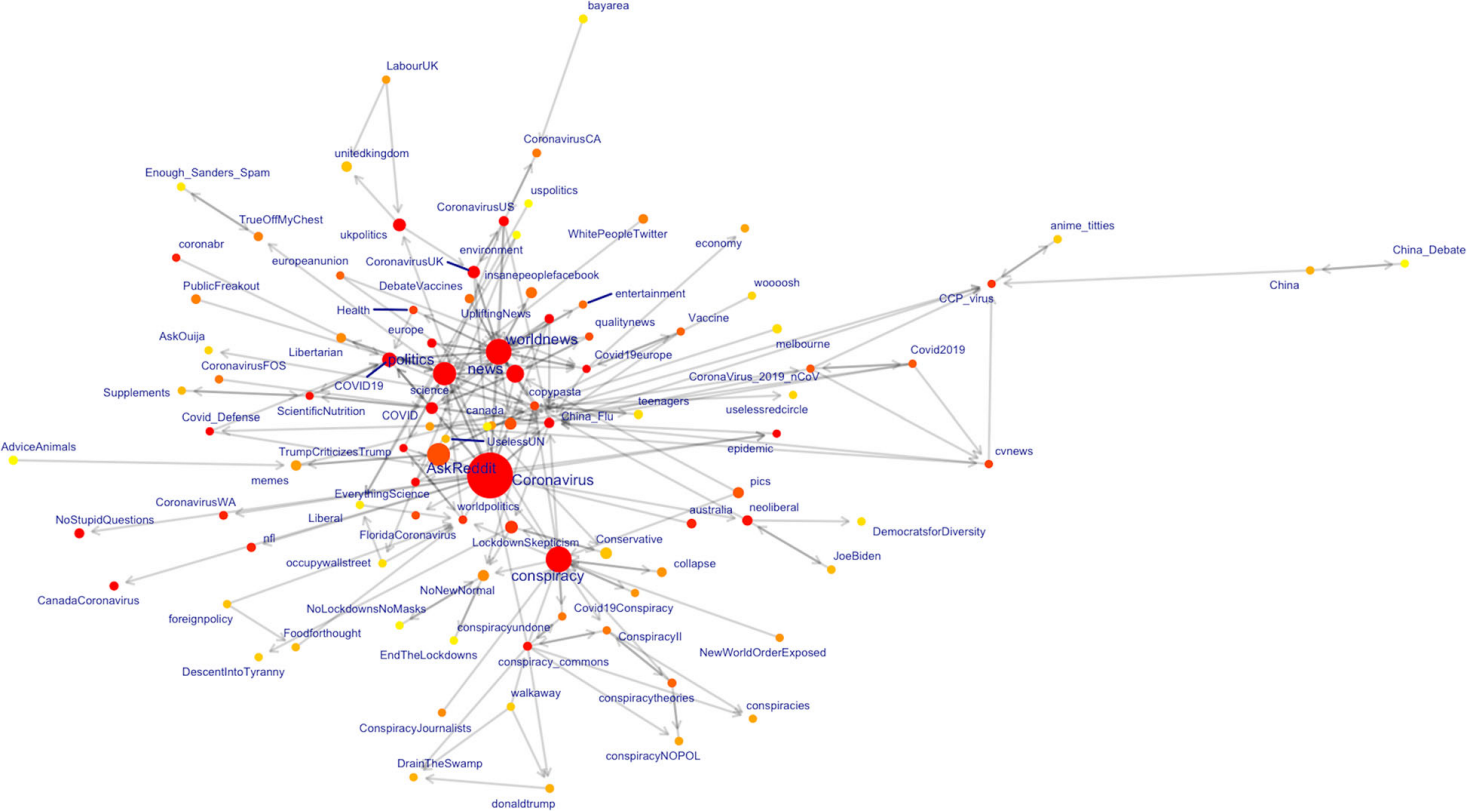
Graph of subreddits that have COVID 19 vaccine-related posts. The largest subgraph above shows subreddits that have COVID-19 vaccine-related posts. Node sizes represent subreddit user sizes, node edges indicate shared COVID-19 vaccine posts between subreddits, and node labels indicate the subreddit name. Node color is based on centrality (red is higher centrality, yellow is lower centrality). Edge direction was based on whether a node had >50% of its posts prior to its adjacent connecting node e.g. A- >B if >50% of A’s shared posts were made before B. We used larger labels for subreddit nodes with >5,000 COVID-19 vaccine posts

To understand the relationship between subreddits that shared COVID-19 vaccine posts, we analyzed the greatest component subgraph, as this was substantially larger than all other subgraphs which had 1-5 nodes and did not provide for meaningful conclusions (see Fig. 2). The largest node/subreddit (r/Coronavirus, the official community for COVID-19 on Reddit) had 2.4 million users. Nodes connected by an edge shared two to 41 posts.

We found nine posts that were first posted in r/Coronavirus and then subsequently posted in at least one subreddit. Posts were reposted one to 10 times. Eight of these posts concerned evidence-based information (e.g. COVID-19 timeline, Vaccine development timeline) and were reposted in other subreddits favoring evidence-based information (e.g. AmericanPolitics, worldnews). However, one post (COVID-19 much milder than believed) was aligned with vaccination skepticism and subsequently posted in subreddits favoring vaccine skeptic narratives (e.g. conspiracy, NoNewNormal - we read through the first 50 posts in these subreddits and verified they were largely around disagreement with evidence-based measures to mitigate the pandemic). This suggests that misinformation is present in some subreddits which generally feature accurate information. This may also indicate that most posts which start in the main COVID-19 subreddit (r/Coronavirus) and then re-posted in other subreddits tend to be evidence-based. However, a minority of posts in r/Coronavirus are skeptical of vaccination, but then do not get reposted in evidence-based subreddits, but instead in subreddits broadly skeptical of vaccination.

## Discussion

Our analysis of 266,840 posts on COVID-19 vaccines between March 11 2020 - December 14 2020 generated several key findings, useful for understanding the early stages of the COVID-19 vaccine timeline. First, there was a relationship between interim positive announcements followed by increased vaccine misinformation, and a relationship between halting vaccine trials and reduced misinformation discussion. Past research has indicated shifts in vaccine perceptions with time [56, 57]. We expand on that work, suggesting an association between events early in the vaccine timeline and vaccine perceptions. Information skeptical of vaccination may flow from a regulated and legitimate source to avenues centering on misinformation and distrust in science. Previous research indicated how antivaccine posts travel online, with users largely moving from one antivaccine post to another [58, 59]. Building on this work, we propose that individuals skeptical of vaccination may selectively highlight posts from legitimate online environments and then forward these posts in arenas aligned with vaccine-skeptic narratives, moving information that was previously under the purview of a more neutral, science-trusting audience to individuals skeptical of vaccination - perhaps providing opportunities to engage such individuals and reduce misinformation. The strength of our work is the use of computational methods to explore how Reddit vaccine perceptions are associated with events early in the vaccine timeline and how posts move among environments with differing vaccine perceptions. Such outcome measurement is central to understanding how vaccine perceptions shift early in the vaccine timeline. Findings may allow for accurate public health messaging when vaccines are first announced, capable of improving COVID-19 vaccine perceptions in the critical initial periods of the vaccine timeline.

There was an association between positive vaccine developments and an increase in discussion of COVID-19 vaccine misinformation, and a relationship between development setbacks and reduced misinformation discussion. Past research has indicated shifting vaccine perceptions over time [57], but there is limited research on specific events, especially early vaccine trials and their relationship with vaccine perceptions. Previous work also indicated that COVID-19 misinformation can be remedied with scientific facts [60], but we highlight the complexity of the phenomenon. The spread and production of misinformation can sometimes be due to confirmation bias, where individuals consume, interpret, and favor information that supports their beliefs [61]. For true antivaxxers, COVID-19 vaccine successes may be interpreted as attempts by Bill Gates to track the population through microchips, and thus news around vaccine successes may be interpreted in a misinformation framework, perhaps explaining the relationship between vaccine success and increased discussion around COVID-19 vaccine conspiracy theories. Similarly, when vaccine trials are halted, such news may cohere with antivaxxers, who may have no interest in engaging with news that possibly demonstrates the failure of medical science - given antivaxxers’ distrust of medical experts [62], perhaps explaining the reduced misinformation discussion. Thus, simply presenting scientific data to antivaxxers [60] may not be effective, as demonstrated in a study where presenting some antivaxxers with facts made them more antivaccine [63].

There was a relationship between an early vaccine trial halting and increased discussion around suspicion of science and mainstream media, and a vaccine trial being effective and reduced discussion around suspicion of science and mainstream media. Factors such as political conservatism and lower levels of education may be associated with lack of trust in science [64], and we build on such research by suggesting that news around science successes and setbacks is associated with trust in science. In an environment where individuals are unsure what to believe around vaccines [65], we propose that early vaccine successes build faith in science, and vaccine setbacks erodes this trust.

We also documented how posts skeptical of vaccination, early in the vaccine timeline, may move from more legitimate avenues to arenas where vaccine-skeptic narratives are more popular. In addition, such posts were popular among some highly active users in our dataset. COVID-19 misinformation is present in mainstream environments and does not always get fact-checked [66] and Reddit is no different. Individuals with largely antivaccine beliefs seek out information that coheres with their views [58]. We build on this work and suggest that individuals skeptical of vaccination, early in the vaccine timeline, also look for information from venues that tend to have evidence-based discussion, but then may interpret such information in line with their views and moral foundations, later sharing such information in forums more skeptical of vaccination. This may indicate that skeptics of vaccination do venture out of their echo chambers to enter spaces where accurate information is the norm - presenting attractive opportunities for constructive intervention.

To improve COVID-19 vaccine perceptions, especially early in the vaccine timeline, minimize misinformation, and increase vaccination rates, public health authorities should conduct tailored interventions and communications campaigns to counter the rhetoric of vaccine misinformation [67, 68]. An example intervention could ask respondents to determine information accuracy around vaccines [69, 70] nudging individuals through the design of these programs toward accurate vaccine information. It is possible that interventions of this sort could shift the beliefs of the vaccine hesitant and thereby boost vaccine uptake, despite potentially little or no effect on committed opponents of vaccination. The concomitant spread of misinformation about COVID-19 vaccines and scientific implications provides insights about the mechanism of misinformation spread. Given our findings around a vaccine trial halting and increased discussion around suspicion of science, we suggest that scientists be more communicative on the difficulties they face in creating vaccines to mitigate science mistrust. Communications campaigns can harness these findings and forward evidence-based posts in subreddits where misinformation is common, when vaccine trial data is released. Given the possibility that individuals seemingly more interested in antivaccine narratives may sometimes venture into more evidence-based environments, interventions can target skeptics or critics of vaccination who sometimes enter more mainstream spaces, engaging them with more evidence-based information, keeping in mind how antivaxxers may deal with such information. Similarly, as legitimate online spaces contain COVID-19 vaccine misinformation, more effective moderation policies can be enacted in these and similar environments e.g. perhaps including a “verified” tag to a post if it comes from a credible source. Such measures may augment health outcomes through several modes. For example, improved vaccine perceptions, especially early in the vaccine development timeline, may lead to reduced vaccine hesitancy and thereby increase vaccine acceptance and COVID-19 vaccination rates. Reduced vaccine misinformation may also improve trust in science and health systems, more broadly, enhancing larger efforts to address health disparities observed in vaccination coverage and many other areas [71].

### Limitations

Our findings relied on the validity of data collected with our search terms. We searched all of Reddit for COVID-19 vaccine posts, and our data contained text fragments representative of vaccine perceptions. We are thus confident in the comprehensiveness of our data. Any use of Reddit data presents several challenges and limitations. As no personal information is collected on Reddit, the demographic makeup of users is unknown [72]. The sample was likely represented by male, younger than general population and mostly based in the US [73]. Thus, the results of the research are affected or influenced by these characteristics of the sample. We note the rapidly changing situation of the COVID-19 pandemic where our data does not reflect the latest situation of the pandemic. We instead provide a cross-sectional overview of the pandemic when vaccine developments were first reported, supplying information stakeholders can utilize for future vaccine roll-outs. We note that the time period of analysis witnessed major US political polarization, major economic shifts in economy, and changes in social lives which may explain some of the variation in our results. Future work will attempt to control for these factors.

It was not possible to determine what posts were viewed by skeptics of vaccination in more legitimate subreddits, but subsequently not reposted in subreddits more supportive of antivaccine narratives, thereby providing more support for our suggestion around confirmation bias. It is possible that posts were made in one subreddit before another purely due to chance, and that the directionality assumed is due to coincidence. We cannot be certain why individuals created the text in our data, the processes behind the shift in narratives, and why individuals shared the same post in more than one subreddit, and we cannot address these mechanisms with our data. Future work can address these questions and explore the motivations of those creating and sharing such text. We conducted a retrospective and observational study, and thus cannot draw causal conclusions regarding vaccine perceptions. It is possible that other vaccine-related events may have caused the observed changes, and that vaccine success stimulated debate that brought to the surface existing antivaccine discussion, instead of causing it.

## Conclusion

Our analysis of Reddit posts on COVID-19 vaccines between March 11 2020 - December 14 2020 provided several key findings, central to understanding the early period of the COVID-19 vaccine timeline. First, we found an association between positive vaccine developments and an increase in discussion of COVID-19 vaccine misinformation, and a relationship between development setbacks and reduced misinformation discussion. We also noted a relationship between an early vaccine trial halting and increased discussion around suspicion of science and mainstream media, and a vaccine trial being effective and reduced discussion around suspicion of science and mainstream media. Finally, we noted how posts skeptical of vaccination, early in the vaccine timeline, may move from more legitimate avenues to arenas where vaccine-skeptic narratives are more popular.

To improve COVID-19 vaccine perceptions, especially early in the vaccine timeline, public health authorities can conduct tailored interventions and communications campaigns to counter vaccine misinformation. Building on our findings around a vaccine trial halting and increased discussion around suspicion of science, we propose that scientists provide more insight on the difficulties around vaccine development. Noting the possibility that individuals seemingly more interested in antivaccine narratives may sometimes venture into more evidence-based environments, interventions can target critics of vaccination in more mainstream spaces, engaging them with more evidence-based information. As the period of our data extends to the period immediately prior to the launch of large-scale US vaccination, stakeholders may use findings to improve future vaccination communication efforts.

## Supplement

### Software

All analysis was conducted using python and R with the following packages: datetime [74], dplyr [75], ggraph [76], grid [77], gridExtra [78], igraph [54], lubridate [79], NumPy [80], pandas [81], pracma [82], praw [83], quanteda [84], readtext [85], readr [86], stm [49], stminsights [87], splines [88], stringr [89], textclean [90], tidygraph [91], tidytext [92], tidyverse [93].

### Search terms

#### COVID-19 keywords

(coronavirus OR coronaviruses OR corona virus OR corona viruses) OR (coronavirus infections OR corona virus infections) OR ’(betacoronavirus OR beta coronavirus OR beta coronaviruses OR betacoronaviruses OR beta corona virus OR beta corona viruses OR betacorona virus OR betacorona viruses) OR (severe acute respiratory syndrome coronavirus OR severe acute respiratory syndrome corona virus) OR SARS CoV-2 OR cov2 OR sars 2 OR COVID OR (coronavirus 2 OR corona virus 2) OR covid19 OR nCov OR (new coronavirus OR new corona virus) OR (novel coronavirus OR novel corona virus) OR (novel coronavirus pneumonia OR novel corona virus pneumonia) OR ncp OR (pneumonia AND (wuhan|china|chinese|hubei))

#### Vaccine keywords

(vaccine OR vaccinate OR vaccinated OR vaccinating OR vaccines OR vaccinates OR vaccination OR vaccinations) OR (immunisation OR immunise OR immunising OR immunisations OR immunises OR immunised) Or (immunization OR immunizations OR immunize OR immunized OR immunizes OR immunizing)

### Online news sites

historyofvaccines.org/content/articles/coronavirustimelineimmunize.org/timeline biospace.com/article/a-timeline-of-covid-19-vaccine-development fortune.com/2020/12/30/covid-vaccine-first-coronavirus-cases-timeline-2020

### Vaccination experts

We identified key scholars in vaccination through the number of articles (>10) published regarding vaccination. We then contacted the identified researchers and asked them to assist.

### Longlist of vaccine-related events

Fauci says he is cautiously optimistic that a vaccine will be effective and achieved within 1 or 2 years (May 12 2020)United States and AstraZeneca Form Vaccine Deal (May 21 2020)Moderna Vaccine Begins Phase 3 Trial, Receives $472M From then-US President Donald Trump’s Administration (July 27 2020)AstraZeneca Halts Phase 3 Vaccine Trial (September 8 2020)Johnson & Johnson Halts Vaccine Trial (October 12 2020)Pfizer announcing preliminary vaccine clinical trial results showing 90% efficacy (November 9 2020)

### Topic modeling

Within topic modeling, a topic is a distribution over a vocabulary [94]. For example, in a topic denoted “vape”, there is likely a greater probability that the terms “smoke” and “device” occur than the words “peanut” and “tomato”. “Smoke” may appear in both “vape” and “cooking” topics with different contextual meanings. Given the topic is a distribution, “smoke” may appear with other high-probability terms like “roast” and “fry” in the “cooking” topic, but with terms like “nicotine” and “device” in the “vape” topic. Thus, topics can be understood as if a person was to talk about a topic and when doing so, tended to use some words than others when the topic is “cooking” compared to “vape”. Topic models are apt for analyzing large quantities of textual data via an automated technique for providing context.

The key innovation of STM is that it can incorporate metadata or information about each document. We thus used STM instead of other topic modelling techniques as STM can incorporate covariates central to our topic of interest [49]. This allows metadata covariates, such as new COVID-19 cases per day, to influence topic discovery. Metadata can affect both topic prevalence and content. Metadata covariates for topical prevalence allow the metadata to affect topic frequency. Similarly, covariates in topical content allow the metadata to affect the word rate within a topic or how a topic is discussed [49]. The STM process will output documents and vocabulary for analysis [49]. Output can be investigated in a range of ways, such as detailing words associated with topics or the relationship between metadata and topics. Model output can be used to conduct hypothesis testing around these relationships.

The number of topics was based on our understanding of the dataset and how other researchers interpreted STM results [52, 95]. Choosing the number of topics was also influenced by post-estimation validation outcomes and past work [52]. As per standard content analysis [96], topic model validation also needs qualitative review, where researchers assess the interpretability and relative efficacy of models based on their subject matter expertise and data context. Our final model [k=20] provided the greatest external validity and most semantically coherent output of distinctive topics. Above the indicated number of topics, there were diminishing returns for solutions, as the substantive meaning and coherence of categories started to break down. Below the indicated number of topics, variation decreased and specific topics got placed into more generic categories. Validating a topic model is not the same as evaluating a statistical model regarding a population sample [97]. The goal is to identify the framework which best describes the data, not estimating population parameters [97].

Most of the text was produced and consumed by people who were interested in the COVID-19 vaccine, and this lens was used to interpret the presence/absence of topics and words. Most of the topic labels were straightforward and did not require much interpretation. To characterize topics in the COVID-19 vaccine narrative, we qualitatively coded each topic by investigating word clouds based on each topic and reviewing exemplar documents which detailed high proportions of each topic [94]. The topic we classified as “Economy and markets” had the following most frequently occurring words: market, company, stock, product, industry, supply, price, sell, demand, billion, economy, trade, million, invest, high. Exemplar documents which exhibited high proportions of this topic indicated a preoccupation with these words. Thus, the interpretation of the topic was clear, given the genre of the narrative and relying on research regarding prominent topics around the COVID-19 vaccine.

Topic validation is key to assessing whether the substantive meaning of the topic and its words are parallel with the qualitative meaning of the text and we used methodological guidance from past research for this purpose [47, 94]. Past work advocated the use of sample documents to validate each topic’s substantive meaning. Determining the number of sample documents to use is based on the amount of resolution needed by a social scientist to answer the research question using topic modeling methods [98]. Thus, determining the number of sample documents is a largely qualitative process, dependent on the research question at hand. To determine the appropriate number of documents to sample, we searched the social science literature for studies that used topic modeling, based on the following study characteristics: 1) similar research questions as our study; 2) similar topic areas as our study; 3) study data was drawn from similar sources as our study. We searched databases such as Web of Science Core Collection, Embase, PsycINFO, MEDLINE and Sociological Abstracts. We used keywords such as vaccine, misinformation, and topic modeling. The paper by Farrell (2016) was determined to be most similar to our study based on the assessed characteristics. Farrell (2016) explored ideological polarization in climate change and used a broad range of sources, such as press releases, published papers, and website articles. Based on the nature of the research question and large range of sources, Farrell (2016) determined that a sample of 50 documents was sufficient to validate the substantive meaning of the topic output. Given the similarities between Farrell’s (2016) study and ours on a range of characteristics, we similarly determined that a sample of 50 documents was adequate to validate the topics. We used findThoughts and plotQuote within the STM package to examine the top 50 associated documents for each topic to validate a topic’s substantive meaning. Determination of the top 50 documents was based on ranking topics by the maximum a posteriori estimate of the topic’s theta value, which represents the modal estimate of the proportion of word tokens assigned to the topic with the model. These top 50 documents were read by two of the authors to determine validity (k >0.8). A third author resolved disagreements where necessary.

## Data Availability

The datasets used and analyzed during the current study available from the corresponding author on reasonable request. The data used in the study is available at this link: https://osf.io/urp2a/files.
